# Supplementary material for: Towards a Sustainable Reproduction Management of Dairy Sheep: Glycerol-Based Formulations as Alternative to eCG in Milked Ewes Mated at the End of Anoestrus Period
Source: Animals (Basel). 2021 Mar 24;11(4):922. doi: 10.3390/ani11040922 (PMC8064100; doi:10.3390/ani11040922)
Supplement: Supplementary file 1 [file animals-11-00922-s001.pdf]

animals

Supplementary Table 1. Body weight (BW), BCS and their variations (Dif.) during treatment period (between D-13 and D2) in ewes orally drenched with a glycerol-based formulation (GLU; *n* = 24) or with water but receiving an eCG administration (GON; *n* = 24). Mean ± SE.

	GLU			GON		
BW1 D−13 (kg)	44.68	±	1.18	44.73	±	0.95
BW2 D2 (kg)	44.69	±	1.25	45.86	±	0.92
Dif BW2-BW1 (kg)	0.01[a]	±	0.46	1.13[b]	±	0.26
BCS1–D-13	2.57	±	0.05	2.56	±	0.04
BCS2–D2	2.48	±	0.05	2.53	±	0.04
Dif. BCS2-BCS1	−0.09	±	0.04	−0.02	±	0.04

Different letters designate difference between group means at $p < 0.05$
